# New Appliances and Things Medical

**Published:** 1904-04-16

**Authors:** 


### NEW APPLIANCES AND THINGS MEDICAL.

[We shall be glad to receive, at our Office, 28 & 29 Southampton Street, Strand, London, W.C., from the manufacturers, specimens of all new preparations and appliances which may be brought out from time to time.]

### PAGET'S MILK FOOD.

(Clay, Paget and Co., Limited, 71 Ebury Street, London, S.W.)

We have on more than one occasion insisted that some form of suitably modified cow's milk is the ideal food for those infants who, for some reason or other, cannot be given the breast. A short while ago we described a method of "humanising" cow's milk at home. To those who find this process too tedious or difficult we can recommend Paget's Milk Food. The preparation is a concentrated and modified cow's milk. When mixed with water (the latter preferably boiled beforehand) in the proportion of two of the latter to one of the food, the product will be practically identical in its percentage composition with human milk. From this it will be seen that a perfect substitute for maternal food can be supplied with a minimum amount of trouble. One important point needs to be mentioned. The Milk Food is prepared at the company's laboratory at Hounslow from perfectly fresh cow's milk immediately after milking; further, it contains no artificial preservative. The Food is supplied in 5 oz. and 10 oz. bottles, the price being 7s. and 12s. per dozen. So long as the bottles are not opened, the contents remain sterile.

